# A Unified Current-Voltage Model for Metal Oxide-Based Resistive Random-Access Memory

**DOI:** 10.3390/ma16010182

**Published:** 2022-12-25

**Authors:** Harry Chung, Hyungsoon Shin, Jisun Park, Wookyung Sun

**Affiliations:** 1 Department of Electronic and Electrical Engineering, Ewha Womans University, Seoul 03760, Republic of Korea; 2 Graduate Program in Smart Factory, Ewha Womans University, Seoul 03760, Republic of Korea; 3 Department of Electrical and Computer Engineering, Seoul National University, Seoul 08826, Republic of Korea

**Keywords:** resistive random-access memory (RRAM), resistive switching, memristor, memristive device, HSPICE

## Abstract

Resistive random-access memory (RRAM) is essential for developing neuromorphic devices, and it is still a competitive candidate for future memory devices. In this paper, a unified model is proposed to describe the entire electrical characteristics of RRAM devices, which exhibit two different resistive switching phenomena. To enhance the performance of the model by reflecting the physical properties such as the length index of the undoped area during the switching operation, the Voltage ThrEshold Adaptive Memristor (VTEAM) model and the tungsten-based model are combined to represent two different resistive switching phenomena. The accuracy of the I-V relationship curve tails of the device is improved significantly by adjusting the ranges of unified internal state variables. Furthermore, the unified model describes a variety of electrical characteristics and yields continuous results by using the device’s current-voltage relationship without dividing its fitting conditions. The unified model describes the optimized electrical characteristics that reflect the electrical behavior of the device.

## 1. Introduction

Various conceptual non-volatile memory devices have emerged as competitive candidates for use as next-generation memory devices. Among them, resistive random-access memory (RRAM) is one of the most competitive future memory device candidates, and therefore, RRAM and its applications have been researched extensively. RRAM consists of metal–insulator–metal (MIM) structures and exhibits non-volatile behavior characterized by resistance change and transitions between the low-resistance state (LRS) and high-resistance state (HRS) [1]. Several mechanisms can be used to explain this switching operation such as the valence change mechanism (VCM), electrochemical metallization (ECM), and thermal-chemical mechanism (TCM), all of which are based on the formation of conductive paths called filaments [2,3].

RRAM has mainly been studied from the viewpoint of its use in memory devices owing to its characteristics such as simplicity, scalability, and low-power operation. Recently, its use in neuromorphic devices has also attracted attention [4], because RRAM also satisfies the required attributes from the perspective of fabricating neuromorphic systems [5,6,7,8]. In a neuromorphic system, synapses transfer their signal diversified with multiplying synaptic weight. Diversified signals can be described as continuous and gradual resistance changes in the current-voltage relationship. Therefore, modeling the switching operation of RRAM is still an important issue since it determines the data-storing capability. Furthermore, analog or gradual change in the resistance level, especially after a set or reset processes, has become an essential task to analyze the RRAM operation in neuromorphic system applications.

A unified model of an RRAM device with both switching and analog resistance change operations is proposed herein. The proposed model is an empirical model that improves the description of electrical characteristics related to the abrupt switching operation by applying the physical properties of the device to the VTEAM model [9,10,11]. Moreover, the proposed model modifies and standardizes the existing models [12,13] to cover all ranges of device operation from switching operation between LRS and HRS to resistance change after LRS. The electrical characteristics obtained using the proposed models lead to more accurate switching operations and provide the combined effects of different resistive switching operations simultaneously.

## 2. Materials and Methods

### 2.1. Device Fabrication and Characteristics

**Figure 1:**
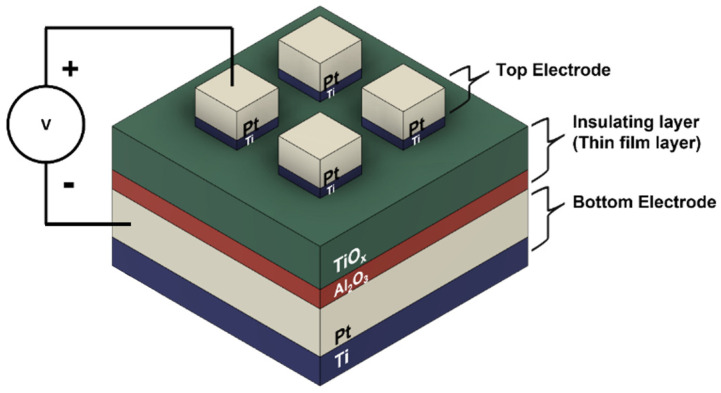
A simple structure of fabricated RRAM device.

A Pt/Ti/TiO_x_/Al_2_O_3_/Pt/Ti device was fabricated to verify the proposed model in various electronic operations [14]. A P-type (100) SOI (Silicon On Insulator) wafer was used for the device substrate. The top and bottom electrodes were deposited with an identical structure composed of a 100-nm-thick Pt layer and a 10-nm-thick Ti layer as the electrode and adhesion layer, respectively. Both layers were deposited by following a direct current (DC) sputtering process. These thin film layers, which play primary roles in the switching mechanism, were processed in two separate steps. First, a 3-nm-thick Al_2_O_3_ layer was deposited by means of atomic-layer-deposition (ALD). Then, a 40-nm-thick TiO_x_ layer was deposited on this Al_2_O_3_ layer by means of reactive DC sputtering accompanied by oxygen injection. Each device cell was patterned using the photolithography technique, and a simple structure with a 100 µm × 100 µm top electrode was obtained by following the metal lift-off process. The device structure is illustrated in Figure 1. The electrical characteristics of the device were measured using a Keithley 4200 semiconductor characterization system.

In the proposed device, the insulating layer is composed of an oxide material, and the two electrodes are composed of inert metals. The switching mechanism of this device can be assumed as VCM because of the oxide-based insulating layer. Accordingly, the migration of the oxygen vacancies formed in the oxide material is the primary driver of the resistance change operation, and the inert metal electrodes are not involved in this operation [15]. If a voltage bias is applied to the device, an abrupt resistance change occurs after exceeding the device voltage threshold and induces the device to transform its state. When a positive voltage bias that exceeds the device voltage threshold is applied, the device resistance decreases abruptly owing to the formation of a conductive filament and device state transformation from HRS to LRS. [16] By contrast, if a negative voltage bias that exceeds the device’s negative voltage threshold is applied, the device resistance increases owing to the rupture of conductive filaments, leading to a reverse state transformation from HRS to LRS. Furthermore, if consecutive voltage biases with identical polarities are applied, the device resistance changes gradually in the same direction [13].

To fabricate the insulating layer of the proposed device, TiOx and Al_2_O_3_ are used in this work. Each oxide layer plays a different role in device operation [17,18]. The Al_2_O_3_ layer serves as a conductive channel under an applied voltage bias, and filament formation occurs in this layer. It is essential to control the thickness and contamination level of the Al_2_O_3_ layer to facilitate filament formation. In contrast, the TiO_x_ layer is a major source of oxygen vacancies due to its deficiency of oxygen ion content. Oxygen ions migrate under the applied voltage bias, and oxygen vacancies move to the conductive channel to form a conductive path, which accelerates the decrease in device resistance. After the first conductive path is developed, only the end portion of the filament is repeatedly formed and ruptured [19].

### 2.2. Unified Model

The proposed model is based on a few existing models that possess distinct electrical characteristics. The unique electrical characteristics of the RRAM device are as follows. First, voltage-driven switching operation occurs in the device, and therefore, voltage thresholds represent the standard for device operation. Second, the trend of resistance change during device operation is nonlinear owing to the oxide-based insulating layer. Finally, gradual resistance change is achieved through the consecutive application of positive voltage biases. The proposed model should possess these properties.

After device formation, positive and negative voltage sweeps are performed to demonstrate the device switching operation in its entirety. The three different equations used in the unified model to demonstrate this operation exhibit distinct electrical characteristics. Equation (1) pertains to the LRS and HRS regions. In the LRS and HRS regions, the resistance does not vary abruptly. Continual linear or exponential resistance change is the dominant operation in these regions. The conventional equation for describing the LRS and HRS regions with a simple linear shape is as follows [1]:

(1) $$I=V/\{R_{\mathrm{off}}\left(\frac{x}{D}\right)+R_{\mathrm{on}}\left(\frac{1-x}{D}\right)\}$$

The parameters $R_{\mathrm{off}}$ and $R_{\mathrm{on}}$ denote the resistances in the HRS and LRS, respectively. The current output was due to the movement of the x/D. x indicates the undoped region in the insulating layer where the filament repetitively forms and ruptures in the range of x. D is the total length of the insulating layer and used for a normalizing internal state variable x. However, Equation (1) describes only linear device operation. The device used in this work exhibits considerable nonlinearity, which can be attributed to the oxide-based insulating layer. Therefore, the proposed model must be able to describe both nonlinear and linear device operation depending on the applied voltage bias, as follows [9,13]:

(2) $$I={\;w}^{n}\beta \sinh\left(\alpha V\right)+\chi \left(\exp\left(\gamma V\right)-1\right)$$

where α, β, γ, and χ denote fitting parameters, and n is a factor that influences the internal state variable w. The former part of Equation (2) describes a linear procedure because it approximates a hyperbolic sine function as a linear curve in the small-voltage domain, and the latter part of Equation (2) describes an exponential procedure. The device exhibits linear growth in the LRS and exponential growth in the HRS. These two different electrical characteristics of the proposed oxide-based RRAM device can be described using Equation (2).

**Figure 2:**
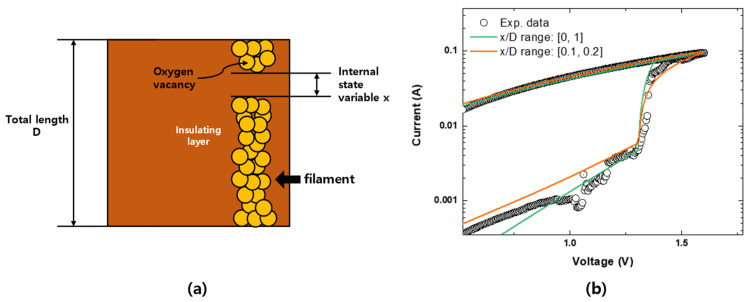
(**a**) Schematic of the rearranged internal state variable x, (**b**) Electrical characteristics of the improved model with reflecting property of the Pt/Ti/TiOx/Al2O3/Pt/Ti device.

In the unified model, the internal state variables of all the constituent models should be rearranged as x normalized with respect to D, where D indicates device length, as explained in Figure 2a. This rearrangement guarantees that separate equations operate under the same criterion. Therefore, with the unified model, the entire resistance-switching operation of the device can be analyzed based on the movement of x/D. Equation (2) is expressed in terms of x/D to obtain Equation (3). Equation (3) is used in the unified model to determine the current-voltage relationships in the LRS and HRS regions of the device. Where N is a fitting parameter for representing the current expression for the LRS. The entire former part of Equation (3), including N, explains the LRS operation.

(3) $$I={\left(1-\frac{x}{D}\right)}^{N}\alpha_{1}\sinh\left(\beta_{1}V\right)+\mathrm{sign}\left(V\right)\alpha_{2}\left[\exp\left(\beta_{2}\left|V\right|\right)-1\right]$$

Equation (4) pertains to the switching region, where the set and reset processes occur. During the HRS to LRS switching operation, the set process accompanied by an abrupt resistance change occurs after the applied voltage exceeds the voltage thresholds of the device. In the switching region, the unified model implemented herein, which is a modified version of the models proposed in [10,11], should be able to set voltage thresholds and cause a large resistance change.

(4) $$\frac{\mathrm{dx}}{\mathrm{dt}}=\left\{\begin{array}{ccc} k_{\mathrm{off}}\cdot {\left(\frac{v\left(t\right)}{v_{\mathrm{off}}}-1\right)}^{\alpha_{\mathrm{off}}} & , & v\leq v_{\mathrm{off}}\\ 0 & , & v_{\mathrm{off}}<v<v_{\mathrm{on}}\\ k_{\mathrm{on}}\cdot {\left(\frac{v\left(t\right)}{v_{\mathrm{on}}}-1\right)}^{\alpha_{\mathrm{on}}}\; & , & v_{\mathrm{on}}\leq v \end{array}\right.$$

In this model, the value of x calculated using Equation (4) contributes only to the device switching rate, which can be controlled by varying the fitting parameters $k_{\mathrm{off}}$, $k_{\mathrm{on}}$, $\alpha_{\mathrm{off}}$, and $\alpha_{\mathrm{on}}$. The k- and α-factors regulate the switching rate multiplicatively and exponentially, respectively. The voltage thresholds of the set and reset processes are controlled by the parameters $v_{\mathrm{off}}$ and $v_{\mathrm{on}}$, respectively.

Equation (5) represents the resistive switching operation within the LRS. During consecutive voltage sweeps after the LRS, the device exhibits a gradual resistance change; during repetitive positive voltage sweeps, the device resistance decreases gradually. A modified version of Chang, T. et al.’s [12] RRAM model for tungsten-oxide-based devices was used to describe this operation in the unified model:

(5) $$\frac{\mathrm{dx}}{\mathrm{dt}}=\lambda \sinh\left(\eta V\right)-\frac{x}{\tau }$$

Equation (5) represents the gradually intensifying pinched-hysteresis operation of the memristive device that can mainly be attributed to the migration of oxygen vacancies. Moreover, it describes the rate of change in the conductive area with respect to the applied voltage bias. The parameters λ and η are fitting parameters that describe the material properties of the device, and τ is the diffusion time constant. Diffusion of oxygen vacancies is additionally considered in the term −x/τ to describe the natural decay process. However, in this work, the diffusion term was not considered.

The window function [9] should be multiplied with Equations (4) and (5) to limit the range of x/D.

(6) $$f_{\mathrm{off}}\left(x\right)=\exp\left[-\exp\left(\frac{x-a_{\mathrm{off}}}{x_{c}}\right)\right]$$

(7) $$f_{\mathrm{on}}\left(x\right)=\exp\left[-\exp\left(-\frac{x-a_{\mathrm{on}}}{x_{c}}\right)\right]$$

Equations (6) and (7) efficiently regulates the operation range of x/D in terms of x_c_, a_on_, and a_off_. The range of x is bounded between a_on_ and a_off_, and the rate of bounding of x is regulated by the parameter x_c_. This restriction implemented by the window function allows for repetitive resistive switching operations under consecutive voltage sweeps.

Therefore, the unified model composed of Equations (3)–(7) describes the resistive switching operation that occurs in the RRAM device in its entirety.

## 3. Results and Discussion

In the experiments conducted herein, two different resistive switching operations of the fabricated device were inferred from its I-V characteristics. The first voltage sweeps after the forming process led to the full resistive switching operation, which was characterized by a significant change in device resistance from the HRS to LRS; subsequently, when the opposite voltage bias was applied, the device returned to the HRS. Under consecutive voltage sweeps with only the positive bias, the device resistance remained in the LRS region after the end of the first voltage sweep, and with subsequent voltage sweeps, the device resistance decreased gradually. The I-V characteristics of the device indicated that as the device resistance decreased, the magnitude of current flowing through the device increased. The execution of two different operations in the same device necessitated the development of a new method for modeling the device switching behaviors. Therefore, we proposed a unified model for RRAM devices that accurately represents the device switching operation and the gradual increase in device current. The VTEAM model and the WO_x_-based model were combined to describe the switching behavior of the proposed device [10,12].

The two aforementioned models have a few identical properties, which allowed us to combine these models. First, both models define their internal state variables as the dividing guidance between the doped and undoped areas of the device, which determines the filament condition. Therefore, the internal state variables can be combined through a few rearrangements to define an equivalent physical index x/D. In the proposed model, x/D denotes the physical index of the undoped region, which can be defined as the ruptured portion of the filament. The x/D-modified versions of the unified model are expressed in Equations (4) and (5). Moreover, the devices used for each of these models were based mainly on oxide materials in which oxygen vacancies significantly influence changes in device resistance. Both models assume VCM as the switching mechanism, and filament formation in an insulating area affects resistance switching in the models [16,20]. The electrical characteristics resulting from the use of oxide material are reflected in both models. Therefore, the performance of these two models can be improved by combining them.

The device performance in the switching region improved when the physical properties of the device were reflected in the model [21]. The x/D range was restricted between 0 and 1 in the existing model. If x/D is defined as 0, conductive path formation has occurred, and current flows through the device, which leads to the set process. By contrast, if x/D is defined as 1, no more conductive paths exist in the device, which induces the reset process. However, in the proposed RRAM device, x/D does not operate inside [0, 1] because, after the first forming process, only the end portion of the filament is ruptured and formed repetitively upon voltage application [19]. Theoretically, the length of the end portion that influences the switching process is assumed to be 10% of the length of the insulation layer. Therefore, the range of x/D should be limited to 10% of the total filament formed to reflect the physical properties of the device. In the proposed model, the x/D range was revised from [0, 1] to [0.1, 0.2] by using Equations (6) and (7). This modification affected device operation in the switching region because the x/D range determines the switching rate of the device. The improved model is illustrated in Figure 2b, where the curve tail of the I-V relationship depicts the device operation more accurately in relation to the regulation of the x/D range.

**Figure 3:**
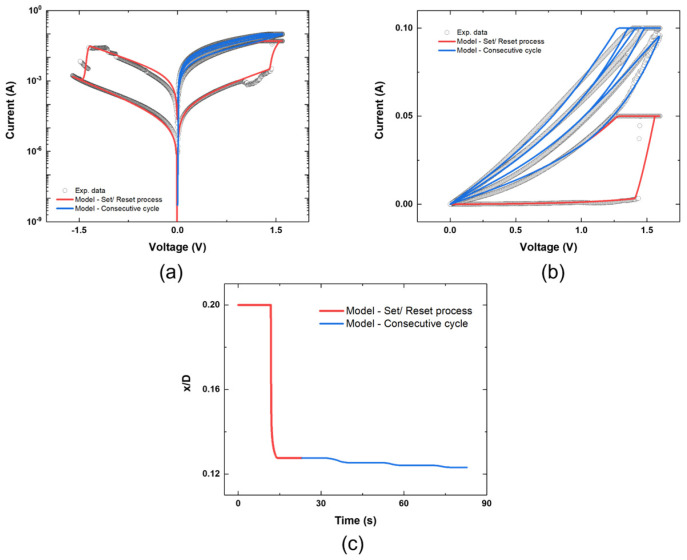
(**a**) Electrical characteristics of the Pt/Ti/TiO_x_/Al_2_O_3_/Pt/Ti device in logscale with the unified model (Sweeping rate = 0.16 V/s), (**b**) Electrical characteristics of the Pt/Ti/TiO_x_/Al_2_O_3_/Pt/Ti device in linear scales with the unified model (Sweeping rate = 0.16 V/s), (**c**) Variation of x/D during the repetitive resistive switching operation represented in Figure 3b.

The unified model exhibited two different resistive switching operations in the experiment. Figure 3 depicts the results obtained using the proposed unified model. The unified model was rearranged using the identically defined internal state variable x/D. As shown in Figure 3a, the unified model comprising Equations (3), (4), (6) and (7) followed the LRS and HRS accurately and caused abrupt resistance changes in the device’s voltage thresholds. Additionally, the results of consecutive voltage sweeps under a positive voltage bias were precisely described using the unified model composed of Equations (3), (5)–(7), as depicted in Figure 3b. Throughout the resistive switching process, the window function introduced in Equations (6) and (7) was implemented in the unified model to control the x/D range. Because the x/D range was bounded, the model was able to exhibit repetitive switching operations. Although the models of each of the voltage sweep cycles originated from different mechanisms, Figure 3a,b describes the entire resistive switching operation within a single device by using the proposed model. The proposed unified model outputs the I-V relationship with undivided parameters for the overall device operation, and the parameters used in the model are listed in Table 1. Moreover, continuous operation can be verified by observing the movement of x/D throughout the resistive switching operation. Figure 3c provides evidence of continuous switching operation in the abrupt resistance-change region and gradual resistance-decrease region. The initial value of x/D is approximately 0.2, and it decreases continuously during the switching process from HRS to LRS and in three consecutive resistive switching cycles.

## 4. Conclusions

In this paper, a unified model was proposed to reflect the entire resistive switching operation of an RRAM device. The RRAM device was fabricated with a Pt/Ti/TiOx/Al2O3/Pt/Ti stacked structure, and it exhibited two separate switching operations with distinct electrical characteristics. The LRS and HRS regions were described using the current-voltage relationship rearranged with respect to the new internal state variable x. The switching operation of the device, accompanied by an abrupt change in device resistance when the voltage applied to the device exceeded its voltage thresholds and was described using an improved model based on the VTEAM model. Moreover, the inaccuracy of the I-V curve tails was solved by considering the physical length index during the switching operation in the model to reestablish the bounds of x/D. The switching operation resulting from the consecutive application of a positive voltage in the LRS region was described using the improved model based on the WO_x_ oxide model. All resistive switching operations were described using the unified model with identical parameters. The movement of x/D verified that the unified model operates continuously and fluently and confirmed that the unified version of the proposed model operates similarly to any of the individual procedures. Therefore, a variety of distinct resistive switching operations of RRAM devices can be described precisely by using the proposed unified model. The unified model with enhanced performance is expected to contribute to the development of numerous RRAM applications, especially neuromorphic device applications, which involve various resistive switching operations.

## Figures and Tables

**Table 1:** Fitting parameters of unified RRAM device model.

Parameter	Value
N	160
A_1_	57,524,968.512
A_2_	1.529 × $10^{-4}$
B_1_	1.350
B_2_	2.204
k_off_	1 × $10^{-5}$
k_on_	−4 × $10^{-2}$
α_off_	3
α_on_	1
v_off_	−1.27
v_on_	1.40
λ	2 × $10^{-9}$
η	3

## Data Availability

Not applicable.
